# Causal Relationship Between Air Pollutants and Blood Pressure Phenotypes: A Mendelian Randomization Study

**DOI:** 10.5334/gh.1404

**Published:** 2025-02-24

**Authors:** Xianshang Zhu, Huabo Mao, Hongyu Zeng, Fengli Lv, Jiancheng Wang

**Affiliations:** 1 First Clinical Medical College, Gansu University of Traditional Chinese Medicine, Lanzhou, Gansu, 730000, China; 2 Department of General Medicine, Gansu Provincial Hospital, Lanzhou, Gansu, 730000, China; 3 Department of Endocrinology, Gansu Provincial Hospital, Lanzhou, Gansu, 730000, China; 4 School of Public Health, Lanzhou University, Lanzhou, Gansu, 730000, China; 5 Gansu health vocational college, Lanzhou, Gansu, 730000, China

**Keywords:** Air pollutants, Blood pressure, Hypertension, Mendelian randomization

## Abstract

**Objectives:**

Hypertension is a chronic disease widely prevalent around the world. While previous observational studies have suggested a link between air pollutants and an increased risk of hypertension, causality has not been established. Our study aimed to investigate potential causal relationships between five air pollutants and four blood pressure phenotypes through two-sample Mendelian randomization.

**Methods:**

Two-sample Mendelian randomization (MR) analyses were performed using genome-wide association studies (GWAS) data from the IEU OpenGWAS project. The main analysis method was the inverse variance weighting (IVW) method. Heterogeneity was assessed by Cochran’s Q test, while pleiotropy was assessed by MR-Egger regression. Sensitivity analysis was performed by weighted median method, MR-Egger method, simple mode method, weighted mode method, and leave-one-out analysis method.

**Results:**

Mendelian randomization results showed positive causal associations between PM10 with hypertension (OR: 1.49; 95%CI: 1.06, 2.09; *P*: 2.23 × 10^–2^) and systolic blood pressure (*β*: 1.89; 95%CI: 0.32, 3.47; *P*: 1.85 × 10^–2^), positive causal associations between PM2.5 and hypertension (OR: 1.26; 95%CI: 1.01, 2.58; *P*: 4.30 × 10^–2^), and negative causal associations between NO_2_ and systolic blood pressure (*β*: –1.71; 95%CI: –3.39, –0.02; *P*: 4.74 × 10^–2^). None of the above associations were subject to pleiotropic bias, and all associations were heterogeneous except for PM10 and hypertension. The leave-one-out analysis showed that no single SNP affected the stability of the results.

**Conclusion:**

Elevated levels of PM2.5 and PM10 have been associated with an increased risk of developing hypertension, with PM10 specifically linked to higher systolic blood pressure levels. Interestingly, NO_2_ has shown potential as a protective factor in lowering systolic blood pressure. This study clarifies the causal relationship between five air pollutants and elevated blood pressure. Ensuring good ambient air quality is essential in preventing hypertension and reducing the overall disease burden.

## 1. Introduction

Air pollution is a significant worldwide issue exacerbated by increased human activity and global industrialization. The presence of air pollutants is a major risk factor for global mortality and morbidity, posing a serious threat to human health (1). In 2015, diseases related to pollution led to around nine million premature deaths, representing 16% of global mortality (2). Without proactive measures, the number of deaths attributed to ambient air pollution is projected to increase by over 50% by the year 2050 (3). Primarily composed of particulate matter and gaseous pollutants, air pollutants often stem from the combustion of solid fuels (4). Particulate matter, with its unique physical properties, can linger in the air for extended periods and travel great distances, potentially causing a range of diseases and significantly reducing human lifespan (4).

Hypertension, a common chronic disease, is a major risk factor for several health conditions including ischemic heart disease, stroke, cardiovascular diseases, chronic kidney disease, and dementia (5). It is a leading cause of cardiovascular disease and premature death worldwide (6). The prevalence of hypertension has been increasing in recent decades and is expected to reach 60% by 2025 (7). Research suggests a decline in hypertension rates in more affluent areas globally, while there is a worrying rise in many low-income regions (8). Factors such as physical activity, diet, environment, and genetics can all play a role in high blood pressure (9). Epidemiological and observational studies have demonstrated significant links between air pollutants and adverse effects on respiratory and cardiovascular health (10). There is substantial evidence supporting the relationship between air pollution and increased blood pressure (11,12). Hypertension remains a major global health issue, particularly in low- and middle-income countries (13). Therefore, further exploration of potential risk factors for hypertension is crucial for effective prevention and management. Despite numerous studies investigating the link between air pollutants and high blood pressure, the results have been inconsistent.

Mendelian randomization (MR) is a widely used method for investigating whether associations are in line with causal theories (14). MR utilizes genetic variants, particularly single nucleotide polymorphisms (SNPs), as instrumental variables for exposure to estimate the causal link between exposure and outcome (15). Since genetic variants are randomly allocated at birth and are not influenced by potential confounders, MR helps address concerns about reverse causation commonly seen in observational studies (16).

Previous Mendelian randomization studies have investigated the causal relationships between five air pollutants and hypertension but have not extensively explored the causal relationships between these pollutants and systolic blood pressure, diastolic blood pressure, and pulse pressure (17). This article seeks to delve into the causal relationship between five air pollutants and various blood pressure phenotypes from a genetic standpoint using the Mendelian randomization method and attempted to analyze the effects of air pollutants on various blood pressure phenotypes, aiming to provide insights into the prevention and management of hypertension and the formulation of related policies.

## 2. Method

### 2.1 Study design

**Figure 1:**
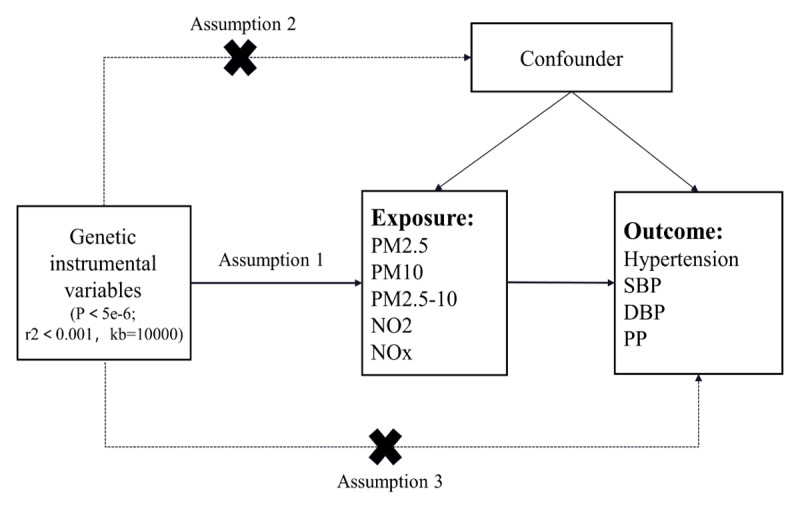
Flow chart of MR Study design. Note: The MR study includes assumptions 1, 2, and 3. The solid line represents a direct causal effect, where the instrumental variable is strongly related to the exposure level and affects the outcome through the exposure. The dashed line indicates that the instrumental variable is not associated with any confounders.

Mendelian randomization studies are based on Mendelian genetic principles and are similar to traditional randomized controlled trial investigations. The use of MR analysis is common in examining causal connections between various exposures and outcomes (18), providing strong causal evidence by utilizing one or more genetic factors, such as SNPs. In our research, we applied a two-sample MR method to explore the potential causal relationship between air pollutants and blood pressure. Figure 1 provides an overview of our study design. The air pollutants considered in this study are PM2.5, PM10, PM2.5–10, NO_2_, and NO_x_. The blood pressure variables of interest encompass essential hypertension, systolic blood pressure, diastolic blood pressure, and pulse pressure. To ensure the credibility of our causal findings, MR analysis needs to meet three key assumptions (19). Firstly, the instrumental variables must demonstrate a strong connection with the exposure factors. Secondly, the instrumental variables must be unaffected by potential confounding variables. Lastly, the instrumental variables should solely impact the results through the exposure under investigation.

### 2.2 Data sources

The air pollution-related factors in this study include PM2.5, PM10, PM2.5–10, NO_2_, and NO_x_. The blood pressure-related factors include hypertension, SBP, DBP, and PP. The GWAS summary-level data for air pollution-related factors were sourced from the UK Biobank via the IEU Open GWAS Project (20), while the data for hypertension were extracted from the FinnGen Biobank. The GWAS data for SBP and DBP were obtained from the International Consortium of Blood Pressure, and the PP data were extracted from a published study, all through the IEU Open GWAS Project (21). Table 1 shows more detailed information about the data sources.

**Table 1:** Genome-wide association study (GWAS) for air pollutants and blood pressure.


GWAS ID	YEAR	TRAIT	SAMPLE SIZE	nSNP	POPULATION

ukb-b-10817	2018	PM2.5	423,796	9,851,867	European

ukb-b-18469	2018	PM10	423,796	9,851,867	European

ukb-b-12963	2018	PM2.5–10	423,796	9,851,867	European

ukb-b-2618	2018	NO_2_	456,380	9,851,867	European

ukb-b-12417	2018	NO_x_	456,380	9,851,867	European

finn-b-I9_HYTENS	2021	Hypertension	218,754	16,380,466	European

ieu-b-38	2018	SBP	757,601	7,088,083	European

ieu-b-39	2018	DBP	757,601	7,160,619	European

ebi-a-CST90018970	2021	PP	360,863	19,047,322	European


### 2.3 Selection of instrumental variables

We first set the threshold for genome-wide correlation to *P* < 5e-8, but this produced fewer instrumental variables. To maintain an adequate number of instrumental variables (IVs) in Mendelian randomization (MR) analysis, a threshold of *P* < 5e-6 is set to select reliable IVs. This criterion has been utilized in previous MR studies (17).

To address the issue of linkage disequilibrium (LD) among instrumental variables, we established thresholds of r^2^ < 0.001 and kb = 10000. The R package ‘TwoSampleMR’ was used to identify independent instrumental variables (IVs). To reduce weak instrumental variable bias and ensure reliable findings, the F value ((R2/K)/[(1-R2)(NK-1)]) was calculated, and IVs with F statistics >10 were excluded. A total of 48 SNPs were identified as strongly associated with PM2.5, 24 SNPs with PM2.5–10, 29 SNPs with PM10, 105 SNPs with NO_2_, and 75 SNPs with NO_x_.

### 2.4 Mendelian randomization analysis

This Mendelian randomization study utilized inverse variance weighting, MR-Egger, weighted median model, simple model, and weighted model to investigate the causal relationship between five air pollutants and four blood pressure phenotypes (22). The main analysis method employed was inverse variance weighting to assess the causal link between exposure and outcome. The other four methods served as supplementary tools to validate the IVW analysis findings and enhance the precision of the research outcomes. The IVW utilizes meta-analysis to merge Wald estimates for each SNP to derive an overall estimate of the impact of exposure on outcomes (23). In this study, the random effects model of IVW was selected over the fixed effects model because of its more conservative approach (24).

### 2.5 Sensitivity analysis

To improve the accuracy of estimates from Mendelian randomization studies, we performed sensitivity analyses. The heterogeneity of instrumental variables was assessed using the Cochran Q test (25). MR-Egger intercept (26) and MR-PRESSO (27) were used to evaluate potential horizontal pleiotropy effects in our MR analysis. In the presence of horizontal pleiotropy, MR-PRESSO was utilized to detect and eliminate outlier SNPs to enhance the robustness of the findings. We employed the leave-one-out method to assess the impact of individual SNPs on the MR analysis results, particularly focusing on SNPs with higher pleiotropy levels. By iteratively removing SNPs and conducting a meta-analysis on the remaining SNPs, we calculated MR results using all remaining SNPs and compared them with results using all SNPs. Any significant changes in the MR results upon excluding an SNP suggest a direct relationship between the SNP and the outcome, potentially violating the assumption that the IV influences the outcome solely through exposure.

### 2.6 Statistical analysis

Statistical analyses were performed using the software packages ‘TwoSampleMR’ (28) and ‘MR-PRESSO’ (27) in R version 4.3.3 (R Foundation for Statistical Computing, Vienna, Austria), with significance defined as *P* < 0.05.

## 3. Results

### 3.1 Single nucleotide polymorphism selection and validation

After a series of filtering processes, we identified 48, 29, 24, 105, and 75 instrumental variables for PM2.5, PM10, PM2.5–10, NO_2_, and NO_x_. Furthermore, to mitigate the potential impact of weak instrumental variables, we ensured that all selected instrumental variables had F values above 10, indicating minimal risk of weak instrumental variable bias affecting the causal effect.

### 3.2 The cause-and-effect relationship between air pollutants and blood pressure

#### 3.2.1 The causal relationship between air pollutants and hypertension

**Figure 2:**
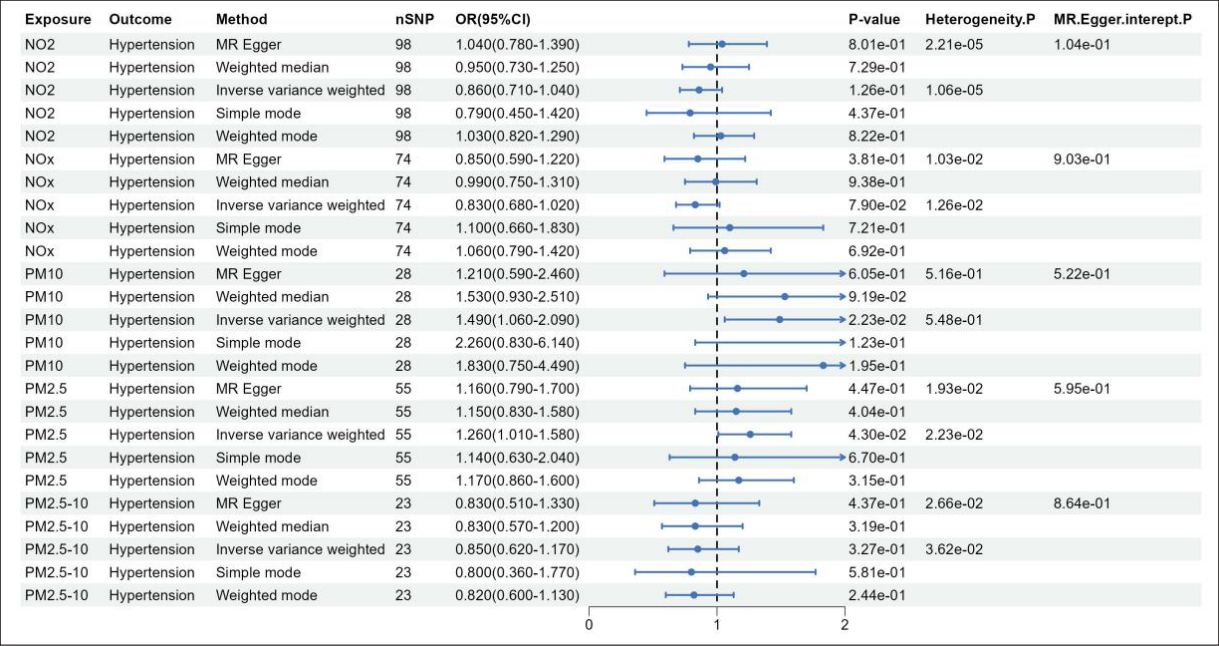
Forest plot of the association between air pollution and hypertension.

The study investigated the causal relationships between five air pollutant components and hypertension. The IVW analysis results revealed the odds ratio (OR) and 95% confidence interval (CI) for each exposure component: PM2.5: OR = 1.26, 95%CI = 1.01 ~ 1.58, *P* = 4.30 × 10^–2^; PM2.5–10: OR = 0.85, 95%CI = 0.62 ~ 1.17, *P* = 3.27 × 10^–1^; PM10: OR = 1.49, 95%CI = 1.06 ~ 2.09, *P* = 2.23 × 10^–2^; NO_2_: OR = 0.86, 95%CI = 0.71 ~ 1.04, *P* = 1.26 × 10^–1^; NO_x_: OR = 0.83, 95%CI = 0.68 ~ 1.02, *P* = 7.90 × 10^–2^ (Figure 2). The IVW method indicates a potential causal relationship between PM2.5 and PM10 exposure and hypertension. However, other methods such as MR-Egger, weighted median, simple model, and weighted model did not provide support for the association between PM2.5, PM10, and hypertension. These findings suggest that higher levels of PM2.5 and PM10 exposure are associated with an increased risk of hypertension, with a 26% and 49% increase in risk, respectively. Furthermore, this study did not find any evidence supporting a causal relationship between PM2.5–10, nitrogen dioxide, nitrogen oxides, and the risk of hypertension.

#### 3.2.2 The causal relationship between air pollutants and systolic blood pressure, diastolic blood pressure, and pulse pressure

**Figure 3:**
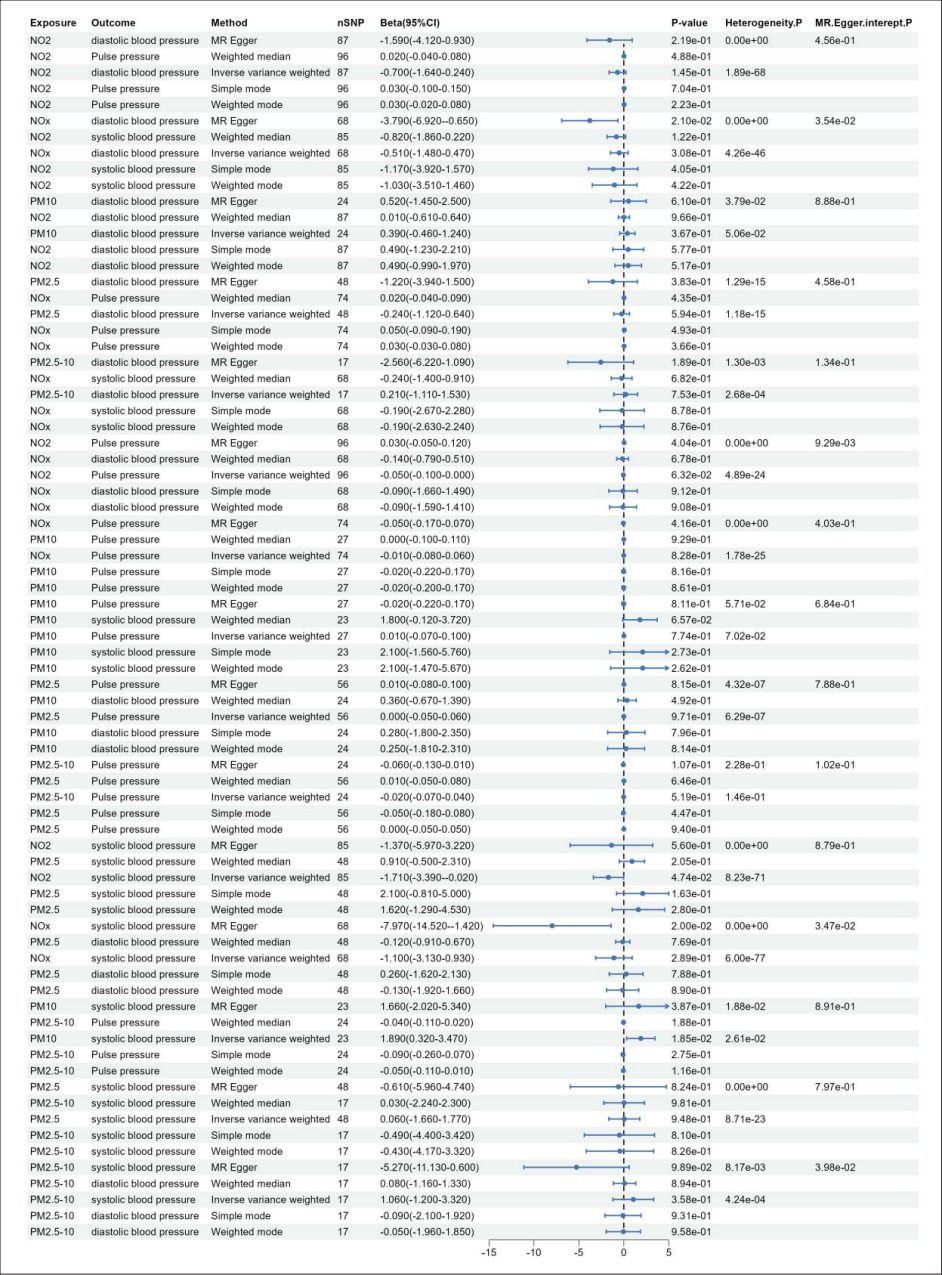
Forest plot of the association between air pollution and systolic blood pressure, diastolic blood pressure, and pulse pressure.

In the causal relationship analysis between five air pollutant components and systolic blood pressure, diastolic blood pressure, and pulse pressure, it was observed that there is a causal relationship between PM10, NO_2_, and systolic blood pressure. The IVW analysis results revealed the associations: PM10: *β* = 1.89, 95%CI = 0.32 ~ 3.47, *P* = 1.85 × 10^–2^; NO_2_: *β* = –1.71, 95%CI = –3.39 ~ –0.02, *P* = 4.74 × 10^–2^ (Figure 3). Our study indicates that elevated levels of PM10 may lead to an increase in systolic blood pressure, whereas higher concentrations of NO_2_ could potentially lower systolic blood pressure and serve as a protective factor against elevated levels of systolic blood pressure. However, no causal relationship was found between PM2.5, PM2.5–10, nitrogen oxides, and systolic blood pressure, and no evidence supported a causal link between the five air pollutants and diastolic blood pressure.

### 3.3 Evaluation of assumptions and sensitivity analyses

In the MR analysis of air pollutants and hypertension, the results of the MR-egger intercept test were not significant for horizontal pleiotropy in the significant causal relationships (Figures 2 and 3), indicating that the selected SNP would not affect the causal relationships through other biological pathways. Among the significant causal relationships, there was heterogeneity in all of them except only PM10 and hypertension (Figures 2 and 3). Despite this heterogeneity, the study used an IVW random effects model to ensure the reliability of the MR results.

**Figure 4:**
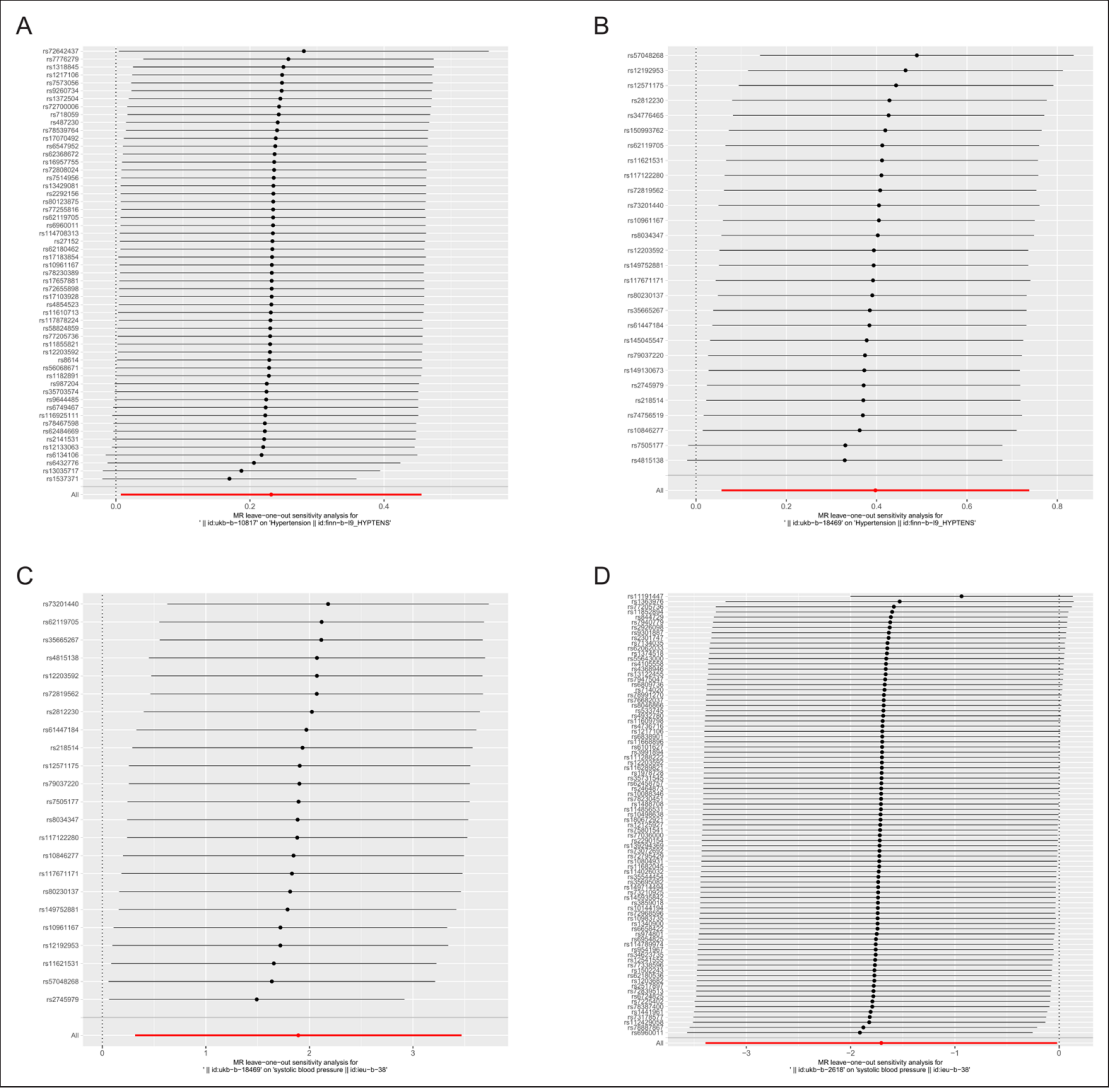
Forest plot of leave-one-out analysis of SNP causal effects of air pollution on blood pressure. Note: Error bars represent 95% confidence intervals (CI). **A:** PM10-Hypertension; **B:** PM10-systolic blood pressure; **C:** PM2.5-hypertension; **D:** NO_2_-systolic blood pressure.

Sensitivity analysis was conducted on Mendelian randomization (MR) outcomes to evaluate the influence of instrumental variables. When investigating the effects of PM10 on hypertension and systolic blood pressure, it was observed that all IVs were situated to the right of zero. When investigating the effects of PM2.5 on hypertension, it was observed that all IVs also were situated to the right of zero. But in the examination of NO_2_ impact on systolic blood pressure, the IVs were positioned to the left of zero. The removal of each SNP did not yield noteworthy alterations in the MR outcomes (Figure 4). These results suggest that the MR findings are robust, implying that PM2.5 and PM10 might be implicated as risk factors for hypertension, while NO_2_ could potentially act as a protective factor against increases in systolic blood pressure. Moreover, the analysis of other exposures revealed that deleting each SNP individually had minimal impact on the outcomes, indicating that no single SNP significantly influenced the overall causal relationship.

## 4. Discussion

### 4.1 Major findings

In this two-sample MR analysis, IVW results indicated a positive causal relationship between PM2.5 (OR: 1.26; 95%CI: 1.01, 2.58; *P*: 4.30 × 10^–2^) and PM10 (OR: 1.49; 95%CI: 1.06, 2.09; *P:* 2.23 × 10^–2^) and hypertension. These results indicated that each 1ug/m3 increase in PM2.5 concentration was associated with a 26% increase in the risk of developing hypertension, whereas a 1ug/m3 increase in PM10 concentration was associated with a 49% increase in the risk of developing hypertension. In addition, the IVW methodology identified a causal relationship between PM10, NO_2_, and systolic blood pressure. Higher PM10 levels were causally associated with increased systolic blood pressure (*β*: 1.89; 95%CI: 0.32, 3.47; *P*: 1.85 × 10^–2^), while higher NO_2_ levels (*β*: –1.71; 95%CI: –3.39, –0.02; *P*: 4.74 × 10^–2^) were causally associated with decreased systolic blood pressure. These findings suggest that each 1ug/m3 increase in PM10 concentration was associated with a 1.89 mmHg increase in systolic blood pressure, whereas a 1ug/m3 increase in NO_2_ concentration was associated with a 1.71 mmHg decrease in systolic blood pressure. These associations were free of pleiotropic bias. The association between PM10 and hypertension was not subjected to heterogeneity bias, which was present in the other associations. Results of leave-one-out analysis showed stable results. No other causal associations were found between air pollutants and hypertension, systolic, diastolic, and pulse pressure.

#### 4.1.1 Causal relationship between PM2.5 and hypertension

This study found a positive causal relationship between PM2.5 and hypertension. Previous studies have indicated a significant positive correlation between long-term exposure to PM2.5 and the onset and prevalence of hypertension (11). Elevated concentrations of PM2.5 are associated with an increased risk of developing hypertension. Our findings confirm that for each standard deviation increase in PM2.5 concentration, there is a 26% increase in the risk of hypertension. A systematic review demonstrated that long-term exposure to air pollutants has varying effects on blood pressure compared to short-term exposure (29). For example, Long-term exposure to PM2.5 is significantly associated with hypertension and diastolic blood pressure, whereas short-term exposure to PM2.5 primarily impacts systolic blood pressure. A recent meta-analysis demonstrated that brief exposure to PM2.5, PM10, and NO_x_ consistently leads to a heightened risk of hypertension. Furthermore, prolonged exposure to PM2.5 is notably linked to hypertension, atherosclerosis, and sudden myocardial infarction (1).

#### 4.1.2 Causal relationship between PM10 and hypertension

Moreover, PM10 was found to have a positive causal relationship with hypertension. A cross-sectional study conducted in South Korea found a positive association between the annual average concentration of PM10 and hypertension (OR = 1.30, 95%CI = 1.12 ~ 1.52). The relationship between PM10 and hypertension was observed to vary based on abdominal fat distribution (30). Additionally, obesity may exacerbate the association between air pollutants and high blood pressure. Research from a cohort study indicates that individuals who are obese are particularly vulnerable to the impacts of air pollution, increasing their risk of developing hypertension (31). This heightened susceptibility could be attributed to the accumulation of visceral fat, which triggers an inflammatory response and damages the endothelium, ultimately contributing to the hypertensive state. Numerous studies indicate that obesity could potentially be a factor in the relationship between air pollution and hypertension. Past research using Mendelian randomization techniques has indicated that the heightened risk of essential hypertension linked to PM2.5 might be influenced by BMI and that BMI may also mediate the impact of PM10 on hypertension (17). Our research findings indicate that PM10 may have a more significant effect on hypertension compared to PM2.5. It is noteworthy that an increase in PM10 concentration appears to primarily elevate systolic blood pressure and contribute to the development of hypertension. While previous observational studies have predominantly examined the influence of PM2.5 on blood pressure, future research should also consider the potential impact of PM10 on blood pressure levels.

### 4.2 Possible mechanisms of elevated blood pressure caused by air pollutants

The mechanisms by which air pollutants cause an increase in blood pressure primarily involve autonomic nervous system dysfunction, oxidative stress, immune-inflammatory response, vascular endothelial dysfunction, and epigenetic changes (32). These factors contribute to arterial vasoconstriction, ultimately leading to elevated blood pressure. However, the specific pathway or mechanism remains unclear. Inflammation is now recognized as a significant pathophysiological factor in hypertension, with various inflammatory markers such as CRP, cytokines, and adhesion molecules found to be elevated in patients with this condition (33,34). Antihypertensive drugs, including diuretics (such as hydrochlorothiazide), beta-blockers (such as carvedilol and bisoprolol), calcium channel blockers, angiotensin-converting enzyme inhibitors, and angiotensin receptor blockers, have been shown to have anti-inflammatory and antioxidant effects while reducing blood pressure (35). A cross-sectional study has demonstrated that air pollutants may elevate blood pressure through the induction of oxidative stress and systemic inflammation. Increasing vitamin C intake has been shown to potentially mitigate the risk of hypertension (36). Furthermore, a separate randomized controlled trial has indicated that melatonin, an antioxidant with fewer side effects, could be advantageous for individuals with essential hypertension by enhancing arteriosclerosis and endothelial function (37). A recent randomized crossover trial demonstrated that air pollutants impact cardiovascular health by modifying human exosomes (38), offering additional support for the connection between air pollutants, epigenetic modifications, and changes in blood pressure. A prospective cohort study indicated a higher prevalence of hypertension among women in regions with air pollution from fossil fuel combustion (39). This underscores the importance of government intervention in safeguarding the health of women, particularly those who may be more vulnerable. The prevalence of hypertension is higher in low- and middle-income countries compared to high-income countries (8). This disparity may be attributed to the increased industrialization in low- and middle-income countries, leading to higher levels of air pollutants. Therefore, there is a pressing need for these countries to prioritize efforts in enhancing air quality to promote better health outcomes for their populations.

The onset and progression of hypertension can be influenced by various factors including social, economic, environmental, and biological factors. Environmental factors, particularly air pollutants, have the potential to impact blood pressure levels through diverse biological pathways (40,41,42). The effects of these mechanisms can vary based on the duration of exposure and individual susceptibility. It is worth noting that the impact of air pollutants on hypertension may be the outcome of the combined influence of multiple pollutants, rather than solely one type of pollutant. Therefore, enhancing air quality and preserving the natural environment remain crucial concerns that should not be overlooked.

### 4.3 Strengths and limitations

This study uses MR methods to explore the causal relationship between air pollutants and high blood pressure. By utilizing genetic variants as exposure IVs, the study improves result accuracy and addresses the issue of reverse causality often seen in observational studies. The inclusion of public GWAS data with a larger sample size enhances the statistical robustness of the findings and offers valuable insights into how different air pollutants impact blood pressure. However, the study’s limitation lies in its focus on individuals of European ancestry, potentially limiting the generalizability of the results to other ethnic groups. Additionally, the use of a significance level of 5e-6 to identify strongly associated SNPs suggests that increasing the sample size could enhance the reliability of the conclusions. While this study provides an initial assessment of the potential links between air pollutants and blood pressure levels, further research is necessary to uncover the specific mechanisms through which air pollutants may contribute to elevated blood pressure.

## 5. Conclusions

In conclusion, this study found a positive causal effect of PM2.5 and PM10 on hypertension and PM10 on systolic blood pressure. Moreover, a negative causal effect between NO_2_ with systolic blood pressure also be found. Our findings may enhance the public’s understanding of the link between air quality and hypertension risk, which is of significant importance for improving current public health policies.

## Additional File

The additional file for this article can be found as follows:

10.5334/gh.1404.s1 Supplementary Material. Supplementary Tables 1–3.
