# Supplementary material for: Causal Relationship Between Air Pollutants and Blood Pressure Phenotypes: A Mendelian Randomization Study
Source: Glob Heart. 2025 Feb 24;20(1):18. doi: 10.5334/gh.1404 (PMC11869833; doi:10.5334/gh.1404)
Supplement: Supplementary Material. — Supplementary Tables 1–3. [file gh-20-1-1404-s1.pdf]

Supplementary Materials

Supplementary Table 1 | Results of Mendelian randomization of air pollutants and blood pressure phenotypes.

**id.exposure**	**id.outcome**	**outcome**	**exposure**	**method**	**nsnp**	**b**	**se**	pval	**lo_ci**	**up_ci**	**or**	**or_lci95**	**or_uci95**
ukb-b-2618	ebi-a-GCST90018970	Pulse pressure	NO2	MR Egger	96	0.03	0.04	4.04E-01	-0.05	0.12	1.04	0.95	1.12
ukb-b-2618	ebi-a-GCST90018970	Pulse pressure	NO2	Weighted median	96	0.02	0.03	4.88E-01	-0.04	0.08	1.02	0.96	1.08
ukb-b-2618	ebi-a-GCST90018970	Pulse pressure	NO2	Inverse variance weighted	96	-0.05	0.03	6.32E-02	-0.10	0.00	0.95	0.90	1.00
ukb-b-2618	ebi-a-GCST90018970	Pulse pressure	NO2	Simple mode	96	0.03	0.07	7.04E-01	-0.10	0.15	1.03	0.90	1.17
ukb-b-2618	ebi-a-GCST90018970	Pulse pressure	NO2	Weighted mode	96	0.03	0.02	2.23E-01	-0.02	0.08	1.03	0.98	1.08
ukb-b-2618	finn-b-I9_HYPTENS	Hypertension	NO2	MR Egger	98	0.04	0.15	8.01E-01	-0.25	0.33	1.04	0.78	1.39
ukb-b-2618	finn-b-I9_HYPTENS	Hypertension	NO2	Weighted median	98	-0.05	0.14	7.29E-01	-0.32	0.22	0.95	0.73	1.25
ukb-b-2618	finn-b-I9_HYPTENS	Hypertension	NO2	Inverse variance weighted	98	-0.15	0.10	1.26E-01	-0.34	0.04	0.86	0.71	1.04
ukb-b-2618	finn-b-I9_HYPTENS	Hypertension	NO2	Simple mode	98	-0.23	0.30	4.37E-01	-0.81	0.35	0.79	0.45	1.42
ukb-b-2618	finn-b-I9_HYPTENS	Hypertension	NO2	Weighted mode	98	0.03	0.11	8.22E-01	-0.20	0.25	1.03	0.82	1.29
ukb-b-2618	ieu-b-38	systolic blood pressure	NO2	MR Egger	85	-1.37	2.34	5.60E-01	-5.97	3.22	0.25	0.00	25.07
ukb-b-2618	ieu-b-38	systolic blood pressure	NO2	Weighted median	85	-0.82	0.53	1.22E-01	-1.86	0.22	0.44	0.16	1.25
ukb-b-2618	ieu-b-38	systolic blood pressure	NO2	Inverse variance weighted	85	-1.71	0.86	4.74E-02	-3.39	-0.02	0.18	0.03	0.98
ukb-b-2618	ieu-b-38	systolic blood pressure	NO2	Simple mode	85	-1.17	1.40	4.05E-01	-3.92	1.57	0.31	0.02	4.81
ukb-b-2618	ieu-b-38	systolic blood pressure	NO2	Weighted mode	85	-1.03	1.27	4.22E-01	-3.51	1.46	0.36	0.03	4.32
ukb-b-2618	ieu-b-39	diastolic blood pressure	NO2	MR Egger	87	-1.59	1.29	2.19E-01	-4.12	0.93	0.20	0.02	2.53
ukb-b-2618	ieu-b-39	diastolic blood pressure	NO2	Weighted median	87	0.01	0.32	9.66E-01	-0.61	0.64	1.01	0.54	1.89
ukb-b-2618	ieu-b-39	diastolic blood pressure	NO2	Inverse variance weighted	87	-0.70	0.48	1.45E-01	-1.64	0.24	0.50	0.19	1.27
ukb-b-2618	ieu-b-39	diastolic blood pressure	NO2	Simple mode	87	0.49	0.88	5.77E-01	-1.23	2.21	1.63	0.29	9.08
ukb-b-2618	ieu-b-39	diastolic blood pressure	NO2	Weighted mode	87	0.49	0.75	5.17E-01	-0.99	1.97	1.63	0.37	7.17
ukb-b-12417	ebi-a-GCST90018970	Pulse pressure	NOx	MR Egger	74	-0.05	0.06	4.16E-01	-0.17	0.07	0.95	0.84	1.07
ukb-b-12417	ebi-a-GCST90018970	Pulse pressure	NOx	Weighted median	74	0.02	0.03	4.35E-01	-0.04	0.09	1.02	0.96	1.09
ukb-b-12417	ebi-a-GCST90018970	Pulse pressure	NOx	Inverse variance weighted	74	-0.01	0.03	8.28E-01	-0.08	0.06	0.99	0.93	1.06
ukb-b-12417	ebi-a-GCST90018970	Pulse pressure	NOx	Simple mode	74	0.05	0.07	4.93E-01	-0.09	0.19	1.05	0.91	1.21
ukb-b-12417	ebi-a-GCST90018970	Pulse pressure	NOx	Weighted mode	74	0.03	0.03	3.66E-01	-0.03	0.08	1.03	0.97	1.09
ukb-b-12417	finn-b-I9_HYPTENS	Hypertension	NOx	MR Egger	74	-0.16	0.19	3.81E-01	-0.53	0.20	0.85	0.59	1.22
ukb-b-12417	finn-b-I9_HYPTENS	Hypertension	NOx	Weighted median	74	-0.01	0.14	9.38E-01	-0.29	0.27	0.99	0.75	1.31
ukb-b-12417	finn-b-I9_HYPTENS	Hypertension	NOx	Inverse variance weighted	74	-0.18	0.10	7.90E-02	-0.39	0.02	0.83	0.68	1.02
ukb-b-12417	finn-b-I9_HYPTENS	Hypertension	NOx	Simple mode	74	0.09	0.26	7.21E-01	-0.42	0.61	1.10	0.66	1.83
ukb-b-12417	finn-b-I9_HYPTENS	Hypertension	NOx	Weighted mode	74	0.06	0.15	6.92E-01	-0.23	0.35	1.06	0.79	1.42

ukb-b-12417	ieu-b-38	systolic blood pressure	NOx	MR Egger	68	-7.97	3.34	2.00E-02	-14.52	-1.42	0.00	0.00	0.24
ukb-b-12417	ieu-b-38	systolic blood pressure	NOx	Weighted median	68	-0.24	0.59	6.82E-01	-1.40	0.91	0.79	0.25	2.49
ukb-b-12417	ieu-b-38	systolic blood pressure	NOx	Inverse variance weighted	68	-1.10	1.04	2.89E-01	-3.13	0.93	0.33	0.04	2.54
ukb-b-12417	ieu-b-38	systolic blood pressure	NOx	Simple mode	68	-0.19	1.26	8.78E-01	-2.67	2.28	0.82	0.07	9.80
ukb-b-12417	ieu-b-38	systolic blood pressure	NOx	Weighted mode	68	-0.19	1.24	8.76E-01	-2.63	2.24	0.82	0.07	9.36
ukb-b-12417	ieu-b-39	diastolic blood pressure	NOx	MR Egger	68	-3.79	1.60	2.10E-02	-6.92	-0.65	0.02	0.00	0.52
ukb-b-12417	ieu-b-39	diastolic blood pressure	NOx	Weighted median	68	-0.14	0.33	6.78E-01	-0.79	0.51	0.87	0.45	1.67
ukb-b-12417	ieu-b-39	diastolic blood pressure	NOx	Inverse variance weighted	68	-0.51	0.50	3.08E-01	-1.48	0.47	0.60	0.23	1.59
ukb-b-12417	ieu-b-39	diastolic blood pressure	NOx	Simple mode	68	-0.09	0.80	9.12E-01	-1.66	1.49	0.91	0.19	4.42
ukb-b-12417	ieu-b-39	diastolic blood pressure	NOx	Weighted mode	68	-0.09	0.77	9.08E-01	-1.59	1.41	0.91	0.20	4.11
ukb-b-18469	ebi-a-GCST90018970	Pulse pressure	PM10	MR Egger	27	-0.02	0.10	8.11E-01	-0.22	0.17	0.98	0.80	1.19
ukb-b-18469	ebi-a-GCST90018970	Pulse pressure	PM10	Weighted median	27	0.00	0.05	9.29E-01	-0.10	0.11	1.00	0.90	1.12
ukb-b-18469	ebi-a-GCST90018970	Pulse pressure	PM10	Inverse variance weighted	27	0.01	0.04	7.74E-01	-0.07	0.10	1.01	0.93	1.10
ukb-b-18469	ebi-a-GCST90018970	Pulse pressure	PM10	Simple mode	27	-0.02	0.10	8.16E-01	-0.22	0.17	0.98	0.80	1.19
ukb-b-18469	ebi-a-GCST90018970	Pulse pressure	PM10	Weighted mode	27	-0.02	0.10	8.61E-01	-0.20	0.17	0.98	0.82	1.19
ukb-b-18469	finn-b-I9_HYPTENS	Hypertension	PM10	MR Egger	28	0.19	0.36	6.05E-01	-0.52	0.90	1.21	0.59	2.46
ukb-b-18469	finn-b-I9_HYPTENS	Hypertension	PM10	Weighted median	28	0.43	0.25	9.19E-02	-0.07	0.92	1.53	0.93	2.51
ukb-b-18469	finn-b-I9_HYPTENS	Hypertension	PM10	Inverse variance weighted	28	0.40	0.17	2.23E-02	0.06	0.74	1.49	1.06	2.09
ukb-b-18469	finn-b-I9_HYPTENS	Hypertension	PM10	Simple mode	28	0.81	0.51	1.23E-01	-0.19	1.82	2.26	0.83	6.14
ukb-b-18469	finn-b-I9_HYPTENS	Hypertension	PM10	Weighted mode	28	0.61	0.46	1.95E-01	-0.29	1.50	1.83	0.75	4.49
ukb-b-18469	ieu-b-38	systolic blood pressure	PM10	MR Egger	23	1.66	1.88	3.87E-01	-2.02	5.34	5.25	0.13	208.01
ukb-b-18469	ieu-b-38	systolic blood pressure	PM10	Weighted median	23	1.80	0.98	6.57E-02	-0.12	3.72	6.07	0.89	41.43
ukb-b-18469	ieu-b-38	systolic blood pressure	PM10	Inverse variance weighted	23	1.89	0.80	1.85E-02	0.32	3.47	6.64	1.37	32.08
ukb-b-18469	ieu-b-38	systolic blood pressure	PM10	Simple mode	23	2.10	1.87	2.73E-01	-1.56	5.76	8.16	0.21	317.79
ukb-b-18469	ieu-b-38	systolic blood pressure	PM10	Weighted mode	23	2.10	1.82	2.62E-01	-1.47	5.67	8.16	0.23	290.07
ukb-b-18469	ieu-b-39	diastolic blood pressure	PM10	MR Egger	24	0.52	1.01	6.10E-01	-1.45	2.50	1.68	0.23	12.13
ukb-b-18469	ieu-b-39	diastolic blood pressure	PM10	Weighted median	24	0.36	0.53	4.92E-01	-0.67	1.39	1.44	0.51	4.03
ukb-b-18469	ieu-b-39	diastolic blood pressure	PM10	Inverse variance weighted	24	0.39	0.43	3.67E-01	-0.46	1.24	1.48	0.63	3.46
ukb-b-18469	ieu-b-39	diastolic blood pressure	PM10	Simple mode	24	0.28	1.06	7.96E-01	-1.80	2.35	1.32	0.17	10.53
ukb-b-18469	ieu-b-39	diastolic blood pressure	PM10	Weighted mode	24	0.25	1.05	8.14E-01	-1.81	2.31	1.28	0.16	10.07
ukb-b-10817	ebi-a-GCST90018970	Pulse pressure	PM2.5	MR Egger	56	0.01	0.05	8.15E-01	-0.08	0.10	1.01	0.92	1.11
ukb-b-10817	ebi-a-GCST90018970	Pulse pressure	PM2.5	Weighted median	56	0.01	0.03	6.46E-01	-0.05	0.08	1.01	0.95	1.08
ukb-b-10817	ebi-a-GCST90018970	Pulse pressure	PM2.5	Inverse variance weighted	56	0.00	0.03	9.71E-01	-0.05	0.06	1.00	0.95	1.06
ukb-b-10817	ebi-a-GCST90018970	Pulse pressure	PM2.5	Simple mode	56	-0.05	0.06	4.47E-01	-0.18	0.08	0.95	0.84	1.08
ukb-b-10817	ebi-a-GCST90018970	Pulse pressure	PM2.5	Weighted mode	56	0.00	0.03	9.40E-01	-0.05	0.05	1.00	0.95	1.05
ukb-b-10817	finn-b-I9_HYPTENS	Hypertension	PM2.5	MR Egger	55	0.15	0.19	4.47E-01	-0.23	0.53	1.16	0.79	1.70

ukb-b-10817	finn-b-I9_HYPTENS	Hypertension	PM2.5	Weighted median	55	0.14	0.16	4.04E-01	-0.18	0.46	1.15	0.83	1.58
ukb-b-10817	finn-b-I9_HYPTENS	Hypertension	PM2.5	Inverse variance weighted	55	0.23	0.11	4.30E-02	0.01	0.46	1.26	1.01	1.58
ukb-b-10817	finn-b-I9_HYPTENS	Hypertension	PM2.5	Simple mode	55	0.13	0.30	6.70E-01	-0.46	0.71	1.14	0.63	2.04
ukb-b-10817	finn-b-I9_HYPTENS	Hypertension	PM2.5	Weighted mode	55	0.16	0.16	3.15E-01	-0.15	0.47	1.17	0.86	1.60
ukb-b-10817	ieu-b-38	systolic blood pressure	PM2.5	MR Egger	48	-0.61	2.73	8.24E-01	-5.96	4.74	0.54	0.00	114.44
ukb-b-10817	ieu-b-38	systolic blood pressure	PM2.5	Weighted median	48	0.91	0.72	2.05E-01	-0.50	2.31	2.48	0.61	10.12
ukb-b-10817	ieu-b-38	systolic blood pressure	PM2.5	Inverse variance weighted	48	0.06	0.88	9.48E-01	-1.66	1.77	1.06	0.19	5.90
ukb-b-10817	ieu-b-38	systolic blood pressure	PM2.5	Simple mode	48	2.10	1.48	1.63E-01	-0.81	5.00	8.15	0.45	148.51
ukb-b-10817	ieu-b-38	systolic blood pressure	PM2.5	Weighted mode	48	1.62	1.48	2.80E-01	-1.29	4.53	5.05	0.28	92.35
ukb-b-10817	ieu-b-39	diastolic blood pressure	PM2.5	MR Egger	48	-1.22	1.39	3.83E-01	-3.94	1.50	0.29	0.02	4.46
ukb-b-10817	ieu-b-39	diastolic blood pressure	PM2.5	Weighted median	48	-0.12	0.40	7.69E-01	-0.91	0.67	0.89	0.40	1.96
ukb-b-10817	ieu-b-39	diastolic blood pressure	PM2.5	Inverse variance weighted	48	-0.24	0.45	5.94E-01	-1.12	0.64	0.79	0.33	1.89
ukb-b-10817	ieu-b-39	diastolic blood pressure	PM2.5	Simple mode	48	0.26	0.96	7.88E-01	-1.62	2.13	1.29	0.20	8.45
ukb-b-10817	ieu-b-39	diastolic blood pressure	PM2.5	Weighted mode	48	-0.13	0.91	8.90E-01	-1.92	1.66	0.88	0.15	5.28
ukb-b-12963	ebi-a-GCST90018970	Pulse pressure	PM2.5-10	MR Egger	24	-0.06	0.04	1.07E-01	-0.13	0.01	0.94	0.87	1.01
ukb-b-12963	ebi-a-GCST90018970	Pulse pressure	PM2.5-10	Weighted median	24	-0.04	0.03	1.88E-01	-0.11	0.02	0.96	0.90	1.02
ukb-b-12963	ebi-a-GCST90018970	Pulse pressure	PM2.5-10	Inverse variance weighted	24	-0.02	0.03	5.19E-01	-0.07	0.04	0.98	0.93	1.04
ukb-b-12963	ebi-a-GCST90018970	Pulse pressure	PM2.5-10	Simple mode	24	-0.09	0.08	2.75E-01	-0.26	0.07	0.91	0.77	1.07
ukb-b-12963	ebi-a-GCST90018970	Pulse pressure	PM2.5-10	Weighted mode	24	-0.05	0.03	1.16E-01	-0.11	0.01	0.95	0.90	1.01
ukb-b-12963	finn-b-I9_HYPTENS	Hypertension	PM2.5-10	MR Egger	23	-0.19	0.24	4.37E-01	-0.66	0.28	0.83	0.51	1.33
ukb-b-12963	finn-b-I9_HYPTENS	Hypertension	PM2.5-10	Weighted median	23	-0.19	0.19	3.19E-01	-0.55	0.18	0.83	0.57	1.20
ukb-b-12963	finn-b-I9_HYPTENS	Hypertension	PM2.5-10	Inverse variance weighted	23	-0.16	0.16	3.27E-01	-0.48	0.16	0.85	0.62	1.17
ukb-b-12963	finn-b-I9_HYPTENS	Hypertension	PM2.5-10	Simple mode	23	-0.23	0.41	5.81E-01	-1.03	0.57	0.80	0.36	1.77
ukb-b-12963	finn-b-I9_HYPTENS	Hypertension	PM2.5-10	Weighted mode	23	-0.19	0.16	2.44E-01	-0.51	0.12	0.82	0.60	1.13
ukb-b-12963	ieu-b-38	systolic blood pressure	PM2.5-10	MR Egger	17	-5.27	2.99	9.89E-02	-11.13	0.60	0.01	0.00	1.82
ukb-b-12963	ieu-b-38	systolic blood pressure	PM2.5-10	Weighted median	17	0.03	1.16	9.81E-01	-2.24	2.30	1.03	0.11	9.95
ukb-b-12963	ieu-b-38	systolic blood pressure	PM2.5-10	Inverse variance weighted	17	1.06	1.15	3.58E-01	-1.20	3.32	2.89	0.30	27.72
ukb-b-12963	ieu-b-38	systolic blood pressure	PM2.5-10	Simple mode	17	-0.49	1.99	8.10E-01	-4.40	3.42	0.61	0.01	30.56
ukb-b-12963	ieu-b-38	systolic blood pressure	PM2.5-10	Weighted mode	17	-0.43	1.91	8.26E-01	-4.17	3.32	0.65	0.02	27.66
ukb-b-12963	ieu-b-39	diastolic blood pressure	PM2.5-10	MR Egger	17	-2.56	1.87	1.89E-01	-6.22	1.09	0.08	0.00	2.98
ukb-b-12963	ieu-b-39	diastolic blood pressure	PM2.5-10	Weighted median	17	0.08	0.63	8.94E-01	-1.16	1.33	1.09	0.31	3.78
ukb-b-12963	ieu-b-39	diastolic blood pressure	PM2.5-10	Inverse variance weighted	17	0.21	0.67	7.53E-01	-1.11	1.53	1.24	0.33	4.62
ukb-b-12963	ieu-b-39	diastolic blood pressure	PM2.5-10	Simple mode	17	-0.09	1.03	9.31E-01	-2.10	1.92	0.91	0.12	6.82
ukb-b-12963	ieu-b-39	diastolic blood pressure	PM2.5-10	Weighted mode	17	-0.05	0.97	9.58E-01	-1.96	1.85	0.95	0.14	6.37

Supplementary table 2 | Results of Mendelian randomization level pleiotropy analysis of air pollutants and blood pressure phenotypes.

id.exposure	id.outcome	outcome	exposure	egger_intercept	se	pval
ukb-b-2618	ieu-b-39	diastolic blood pressure	NO2	0.01	0.02	4.56E-01
ukb-b-2618	finn-b-I9_HYPTENS	Hypertension	NO2	0.00	0.00	1.04E-01
ukb-b-2618	ebi-a-GCST90018970	Pulse pressure	NO2	0.00	0.00	9.29E-03
ukb-b-2618	ieu-b-38	systolic blood pressure	NO2	-0.01	0.03	8.79E-01
ukb-b-12417	ieu-b-39	diastolic blood pressure	NOx	0.05	0.02	3.54E-02
ukb-b-12417	finn-b-I9_HYPTENS	Hypertension	NOx	0.00	0.00	9.03E-01
ukb-b-12417	ebi-a-GCST90018970	Pulse pressure	NOx	0.00	0.00	4.03E-01
ukb-b-12417	ieu-b-38	systolic blood pressure	NOx	0.10	0.04	3.47E-02
ukb-b-18469	ieu-b-39	diastolic blood pressure	PM10	0.00	0.02	8.88E-01
ukb-b-18469	finn-b-I9_HYPTENS	Hypertension	PM10	0.00	0.01	5.22E-01
ukb-b-18469	ebi-a-GCST90018970	Pulse pressure	PM10	0.00	0.00	6.84E-01
ukb-b-18469	ieu-b-38	systolic blood pressure	PM10	0.00	0.03	8.91E-01
ukb-b-10817	ieu-b-39	diastolic blood pressure	PM2.5	0.01	0.02	4.58E-01
ukb-b-10817	finn-b-I9_HYPTENS	Hypertension	PM2.5	0.00	0.00	5.95E-01
ukb-b-10817	ebi-a-GCST90018970	Pulse pressure	PM2.5	0.00	0.00	7.88E-01
ukb-b-10817	ieu-b-38	systolic blood pressure	PM2.5	0.01	0.04	7.97E-01
ukb-b-12963	ieu-b-39	diastolic blood pressure	PM2.5-10	0.05	0.03	1.34E-01
ukb-b-12963	finn-b-I9_HYPTENS	Hypertension	PM2.5-10	0.00	0.01	8.64E-01
ukb-b-12963	ebi-a-GCST90018970	Pulse pressure	PM2.5-10	0.00	0.00	1.02E-01
ukb-b-12963	ieu-b-38	systolic blood pressure	PM2.5-10	0.11	0.05	3.98E-02

Supplementary table 3 | Results of a Mendelian randomization heterogeneity test of air pollutants and blood pressure phenotypes.

id.exposure	id.outcome	outcome	exposure	method	Q	Q_df	Q_pval
ukb-b-2618	ebi-a-GCST90018970	Pulse pressure	NO2	MR Egger	284.84	94	4.35E-21
ukb-b-2618	ebi-a-GCST90018970	Pulse pressure	NO2	Inverse variance weighted	306.21	95	4.89E-24
ukb-b-2618	finn-b-I9_HYPTENS	Hypertension	NO2	MR Egger	163.33	96	2.21E-05
ukb-b-2618	finn-b-I9_HYPTENS	Hypertension	NO2	Inverse variance weighted	167.91	97	1.06E-05
ukb-b-2618	ieu-b-38	systolic blood pressure	NO2	MR Egger	556.39	83	3.38E-71
ukb-b-2618	ieu-b-38	systolic blood pressure	NO2	Inverse variance weighted	556.54	84	8.23E-71
ukb-b-2618	ieu-b-39	diastolic blood pressure	NO2	MR Egger	544.60	85	3.41E-68
ukb-b-2618	ieu-b-39	diastolic blood pressure	NO2	Inverse variance weighted	548.20	86	1.89E-68
ukb-b-12417	ebi-a-GCST90018970	Pulse pressure	NOx	MR Egger	274.05	72	2.47E-25
ukb-b-12417	ebi-a-GCST90018970	Pulse pressure	NOx	Inverse variance weighted	276.74	73	1.78E-25
ukb-b-12417	finn-b-I9_HYPTENS	Hypertension	NOx	MR Egger	102.63	72	1.03E-02
ukb-b-12417	finn-b-I9_HYPTENS	Hypertension	NOx	Inverse variance weighted	102.65	73	1.26E-02
ukb-b-12417	ieu-b-38	systolic blood pressure	NOx	MR Egger	513.54	66	1.69E-70
ukb-b-12417	ieu-b-38	systolic blood pressure	NOx	Inverse variance weighted	549.72	67	6.00E-77
ukb-b-12417	ieu-b-39	diastolic blood pressure	NOx	MR Egger	359.40	66	5.85E-42
ukb-b-12417	ieu-b-39	diastolic blood pressure	NOx	Inverse variance weighted	384.53	67	4.26E-46
ukb-b-18469	ebi-a-GCST90018970	Pulse pressure	PM10	MR Egger	37.05	25	5.71E-02
ukb-b-18469	ebi-a-GCST90018970	Pulse pressure	PM10	Inverse variance weighted	37.30	26	7.02E-02
ukb-b-18469	finn-b-I9_HYPTENS	Hypertension	PM10	MR Egger	25.06	26	5.16E-01
ukb-b-18469	finn-b-I9_HYPTENS	Hypertension	PM10	Inverse variance weighted	25.48	27	5.48E-01
ukb-b-18469	ieu-b-38	systolic blood pressure	PM10	MR Egger	36.58	21	1.88E-02
ukb-b-18469	ieu-b-38	systolic blood pressure	PM10	Inverse variance weighted	36.61	22	2.61E-02
ukb-b-18469	ieu-b-39	diastolic blood pressure	PM10	MR Egger	35.09	22	3.79E-02
ukb-b-18469	ieu-b-39	diastolic blood pressure	PM10	Inverse variance weighted	35.12	23	5.06E-02
ukb-b-10817	ebi-a-GCST90018970	Pulse pressure	PM2.5	MR Egger	121.36	54	4.32E-07
ukb-b-10817	ebi-a-GCST90018970	Pulse pressure	PM2.5	Inverse variance weighted	121.52	55	6.29E-07
ukb-b-10817	finn-b-I9_HYPTENS	Hypertension	PM2.5	MR Egger	76.40	53	1.93E-02
ukb-b-10817	finn-b-I9_HYPTENS	Hypertension	PM2.5	Inverse variance weighted	76.81	54	2.23E-02
ukb-b-10817	ieu-b-38	systolic blood pressure	PM2.5	MR Egger	211.50	46	4.53E-23
ukb-b-10817	ieu-b-38	systolic blood pressure	PM2.5	Inverse variance weighted	211.81	47	8.71E-23
ukb-b-10817	ieu-b-39	diastolic blood pressure	PM2.5	MR Egger	166.88	46	1.29E-15
ukb-b-10817	ieu-b-39	diastolic blood pressure	PM2.5	Inverse variance weighted	168.92	47	1.18E-15
ukb-b-12963	ebi-a-GCST90018970	Pulse pressure	PM2.5-10	MR Egger	26.58	22	2.28E-01

ukb-b-12963	ebi-a-GCST90018970	Pulse pressure	PM2.5-10	Inverse variance weighted	30.10	23	1.46E-01
ukb-b-12963	finn-b-I9_HYPTENS	Hypertension	PM2.5-10	MR Egger	35.23	21	2.66E-02
ukb-b-12963	finn-b-I9_HYPTENS	Hypertension	PM2.5-10	Inverse variance weighted	35.28	22	3.62E-02
ukb-b-12963	ieu-b-38	systolic blood pressure	PM2.5-10	MR Egger	31.24	15	8.17E-03
ukb-b-12963	ieu-b-38	systolic blood pressure	PM2.5-10	Inverse variance weighted	41.79	16	4.24E-04
ukb-b-12963	ieu-b-39	diastolic blood pressure	PM2.5-10	MR Egger	36.93	15	1.30E-03
ukb-b-12963	ieu-b-39	diastolic blood pressure	PM2.5-10	Inverse variance weighted	43.12	16	2.68E-04
